# Advanced molecular characterization of enteropathogenic *Escherichia coli* isolated from diarrheic camel neonates in Egypt

**DOI:** 10.14202/vetworld.2021.85-91

**Published:** 2021-01-12

**Authors:** Momtaz A. Shahein, Amany N. Dapgh, Essam Kamel, Samah F. Ali, Eman A. Khairy, Hussein A. Abuelhag, Ashraf S. Hakim

**Affiliations:** 1 Animal Health Research Institute, Agriculture Research Center, Dokki, Giza, Egypt; 2 Department of Microbiology and Immunology, National Research Centre, 33 Bohouth St., Dokki, Cairo, Egypt

**Keywords:** camel, multidrug-resistant *Escherichia coli*, real-time polymerase chain reaction, sequencing, virulence

## Abstract

**Background and Aim:**

Camels are important livestock in Egypt on cultural and economic bases, but studies of etiological agents of camelid diseases are limited. The enteropathogen *Escherichia coli* is a cause of broad spectrum gastrointestinal infections among humans and animals, especially in developing countries. Severe infections can lead to death. The current study aimed to identify pathogenic *E. coli* strains that cause diarrhea in camel calves and characterize their virulence and drug resistance at a molecular level.

**Materials and Methods:**

Seventy fecal samples were collected from diarrheic neonatal camel calves in Giza Governorate during 2018-2019. Samples were cultured on a selective medium for *E. coli*, and positive colonies were confirmed biochemically, serotyped, and tested for antibiotic susceptibility. *E. coli* isolates were further confirmed through detection of the housekeeping gene, *yai*O, and examined for the presence of virulence genes; *tra*T and *fim*H and for genes responsible for antibiotic resistance, *amp*C, *aad*B, and *mph*A. The isolates in the important isolated serotype, *E. coli* O26, were examined for toxigenic genes and sequenced.

**Results:**

The bacteriological and biochemical examination identified 12 *E. coli* isolates from 70 fecal samples (17.1%). Serotyping of these isolates showed four types: O26, four isolates, 33.3%; O103, O111, three isolates each, 25%; and O45, two isolates, 16.7%. The isolates showed resistance to vancomycin (75%) and ampicillin (66.6%), but were highly susceptible to ciprofloxacin, norfloxacin, and tetracycline (100%). The structural gene, *yai*O (115 bp), was amplified from all 12 *E. coli* isolates and *tra*T and *fim*H genes were amplified from 10 and 8 isolates, respectively. Antibiotic resistance genes, *amp*C, *mph*A, and *aad*B, were harbored in 9 (75%), 8 (66.6%), and 5 (41.7%), respectively. Seven isolates (58.3%) were MDR. Real-time-polymerase chain reaction of the O26 isolates identified one isolate harboring *vt*1, two with *vt*2, and one isolate with neither *gene*. Sequencing of the isolates revealed similarities to *E. coli* O157 strains.

**Conclusion:**

Camels and other livestock suffer various diseases, including diarrhea often caused by microbial pathogens. Enteropathogenic *E*. *coli* serotypes were isolated from diarrheic neonatal camel calves. These isolates exhibited virulence and multiple drug resistance genes.

## Introduction

One-humped Arabian camel (*Camelus dromedarius*) livestock is widely distributed in the Middle East; the animal is well adapted to semi-arid and arid environments. In Egypt, camel herds are important semi-domestic animals heavily used in tourist safaris and transportation and for milk and meat [1].

*Escherichia coli* is a Gram-negative rod in *Enterobacteriaceae*. The bacterium is considered an important constituent of gut microbiota in birds and mammals. However, various strains of this species are pathogenic and cause various gastrointestinal disorders, such as diarrhea [2]. In Egypt, diarrheagenic *E. coli* (DEC), especially Shiga toxin-producing *E. coli*, are described in livestock, including calves, sheep, goat, and buffalo calves [3-5]. However, few studies of DEC in camels are available.

Some strains of DEC are public health concerns and may be transmitted from livestock (e.g., camels) to humans. Gastrointestinal infection may cause non-bloody or bloody diarrhea, hemorrhagic colitis (HC), and hemolytic uremic syndrome. These illnesses threaten millions of peoples worldwide, especially in developing countries [6].

Pathogenic *E. coli* diagnosis requires differentiation from non-pathogenic strains that are constitutive components of normal intestinal flora. Such pathotypes are defined by the presence of one or more definable *E. coli* virulence factors and can be identified by advanced molecular and certain conventional methods [7]. Molecular techniques are more rapid and precise for the identification of bacteria compared with conventional phenotypic methods. Multiplex polymerase chain reaction (mPCR) and quantitative real-time PCR (qRT-PCR) are typically used for identification of bacterial species. Further, sequence analysis is an accurate approach for species delineation and epidemiological tracking [8].

Detection of specific genes is fundamental for diagnosis, confirmation, and/or investigation of virulence and antibiotic resistance of *E. coli* isolates; *yaiO* is a housekeeping gene in *E. coli* orphan open reading frame that encodes a protein originally expressed and localized in the outer membrane [9]. *tra*T is a virulence factor and a constituent of conjugative plasmids that encodes a surface protein responsible for entry exclusion [10]. An essential step for initiation and development of enteritis is bacterial attachment to intestinal epithelial cells. *E. coli* attachment is mediated by fimbriae (adhesins) that bind to host cell receptors; the *Fim*H gene is representative [11].

Resistance of microorganism to multiple antimicrobial drugs is a global threat that interferes with and prolongs therapy. Pathogenic *E. coli* strains characteristically harbor antibiotic resistance genes. *Mph*A is responsible for production of macrolide 2′-phosphotransferase I that inactivates 14-ring macrolides, such as erythromycin and oleandomycin [12]. Another drug resistance gene, *Am*pC, encodes AmpC β-lactamase that degrades penicillins [13]. *aadB* is responsible for the production of 2’-aminoglycoside nucleotidyltransferase enzyme that inactivates aminoglycoside antibiotics [14].

Studies of molecular characteristics of *E. coli* isolates from camel herds in Egypt are limited. The current study explores pathogenic *E. coli* among diarrheic camel calves using advanced molecular techniques.

## Materials and Methods

### Ethical approval

As per CPCSEA guidelines, a study involving clinical and postmortem samples does not require the approval of the Institute Animal Ethics Committee.

### Sampling

Seventy fecal samples were collected from neonatal camel calves raised for meat production. Calves were under 6 months of age and suffered from diarrhea. Calves were raised in the Giza Governorate during 2018-2019. Swabs were immediately placed in cooled boxes, transported to the laboratory, and incubated overnight at 37*°*C in trypticase soy broth (Merck KgaA, Darmstadt, Germany).

### Isolation and identification of E. coli

Each sample was streaked onto MacConkey’s agar (Merck, Germany) and incubated for 24 h at 37°C. Lactose-positive colonies were similarly incubated on eosin methylene blue agar (EMB, Merck, Germany). We used two selective media because EMB is used for selection because it is specific for *E. coli*, and MacConkey is for lactose fermentation.

Green colonies with a metallic luster were considered *E. coli*; such colonies were further identified using the API 20E system (BioMérieux, France) following the manufacturer’s instructions to obtain biochemical profiles of isolates.

The isolates were serologically identified by “Seikin” *E. coli* O-antigen diagnostic antisera (polyvalent and monovalent vials, product code; 312001) following the manufacture’s instructions.

### Antibiogram E. coli isolates

*E. coli* isolates were tested for phenotypical susceptibility to frequently used veterinary antibiotics trough disk diffusion method on Mueller-Hinton agar plates (Oxoid, UK) following the guidelines of the Clinical and Laboratory Standards Institute [15]. A panel of 15 antibiotic disks was used: Cefotaxime (30 μg), cefazolin (30 μg), trimethoprim/sulfamethoxazole (25 μg), sulfaprim (50 μg), ampicillin (10 μg), amoxicillin (10 μg), erythromycin (15 μg), vancomycin (15 μg), amikacin (30 μg), streptomycin (30 μg), gentamycin (10 μg), ciprofloxacin (5 μg), norfloxacin (10 μg), cefadroxil (30 μg), and tetracycline (10 μg).

### Molecular diagnosis of E. coli isolates

#### Polymerase chain reaction (PCR)

DNA extraction

DNA was extracted from *E. coli* isolates using a QIAamp DNA Mini kit (Qiagen, Germany, GmbH) with modifications from the manufacturer’s recommendations. Briefly, 200 µL of sample suspension was incubated with 10 µL of proteinase K and 200 µL of lysis buffer at 56°C for 10 min. After incubation, 200 µL of 100% ethanol was added to the lysate. The sample was then washed and centrifuged following the manufacturer’s instructions. Nucleic acid was eluted with 100 µL of elution buffer provided in the kit.

Oligonucleotide primer

Primers used were supplied from Metabion (Germany) (Table-1) [9,16,17].

**Table-1:** Primers sequences, target genes, amplicon sizes, and cycling conditions used for uniplex, diplex, and multiplex PCR.

Target gene	Primers sequences	Amplified segment (bp)	Primary denaturation	Amplification (35 cycles)			Final extension	References

				Secondary denaturation	Annealing	Extension		
Uniplex								
*Escherichia coli yaiO*	R: 5′ ACGCGTGGTTACAGTCTTGCG 3′ 5′ TGATTTCCGTGCGTCTGAATG 3′	115	94°C 5 min.	94°C 30 s	55°C 40 s	72°C 45 s	72°C 10 min	[9]
Diplex								
*Tra*T	GATGGCTGAACCGTGGTTATG CACACGGGTCTGGTATTTATGC	307	94°C 5 min	94°C 30 s	53°C 40 s	72°C 40 s	72°C 10 min	[16]
*fim*H	TGCAGAACGGATAAGCCGTGG GCAGTCACCTGCCCTCCGGTA	508						
Multiplex								
*amp*C	TTCTATCAAMACTGGCARCC CCYTTTTATGTACCCAYGA	550	94°C 5 min	94°C 30 s	55°C 45 s	72°C 45 s	72°C 10 min	[17]
*aad*B	GAGCGAAATCTGCCGCTCTGG CTGTTACAACGGACTGGCCGC	319						
*mph*A	GTGAGGAGGAGCTTCGCGAG TGCCGCAGGACTCGGAGGTC	403						

PCR=Polymerase chain reaction

PCR amplification

Primers were used in a 50 µL PCR reaction containing 25 µL of EmeraldAmp Max PCR Master Mix (Takara, Japan), 1 µL of each primer (20 pmolar), 9 µL of water, and 10 µL of DNA template. The reactions were uniplex (*yaiO*) [9], diplex (*tra*T and *fim*H) [16], and multiplex (*amp*C, *aad*B, and *mph*A) [17] (Table-1) using a Biometra thermal cycler.

Analysis of the PCR products

The products of PCR were separated by electrophoresis in 1.5% agarose gels (Applichem, Germany, GmbH) in ×1 Tris/Borate/ethylenediaminetetraacetic acid buffer at room temperature using gradients of 5 V/cm. Thirty microliters of PCR product was loaded in each gel slot. A 100 bp ladder (Qiagen, Germany, GmbH) was used to determine fragment sizes. The gel was photographed using a gel documentation system (Alpha Innotech, Biometra, Germany), and data were analyzed with computer software. Reference *E. coli* strains ATCC35150 and HB 101 were used as a positive control and *Staphylococcus aureus* ATCC29737 as a negative control.

### RT-PCR

Positive *E. coli* O26 samples were extracted using a Sigma kit following kit instructions. RT-PCR using a MTplexdtec-RT-qPCR Test (Edifici-Quόrum3, Spain) that includes *vt*1 and *vt*2 genes with *vt* specific primers and probes (Table-2) [18]. Signal was collected, and reactions run in an Applied Biosystem StepOne RT-PCR System. FAM fluorogenic cycle threshold of reactions was identified using StepOne™ software (Life Technology, USA). The qualitative detection module analyzed the samples for the presence of *vt*1 and *vt*2 based on fluorescence levels above the background.

**Table-2:** Real-time PCR: Primer sequences, target gene, and amplicon sizes.

Sequence	Target gene	Amplified segment (bp)	References
F(ATAAATCGCCATTCGTTGACTAC) R(AGAACGCCCACTGAGATCATC)	*vt*1	180	[18]
F(GGCACTGTCTGAAACTGCTC) R(TCGCCAGTTATCTGACATTCTG)	*vt*2	255	

PCR=Polymerase chain reaction

### E. coli DNA sequencing

The amplified *tra*T PCR fragments (307 bp) were excised from gels and the DNA extracted using ExoSAP-IT PCR Product Cleanup kit (Affymetrix, USA) following the manufacturerʼs instructions. Purified amplicons were sequenced with an ABI PRISM^®^ BigDyedye™ terminator cycle sequencing kit with AmpliTaq^®^ DNA polymerase on an MJ Research PTC-225 Peltier Thermal Cycler (Applied Biosystems, USA) following manufacturer’s recommendations. DNA sequences were identified by comparison with established sequences in GenBank.

## Results and Discussion

The bacteriological and biochemical examination of the 70 fecal samples showed 12 *E. coli* isolate (17.1%). These results are similar to the findings of Hussni *et al*. [19], who reported a prevalence of enteropathogenic and enterotoxigenic *E. coli* in camel fecal samples in Qatar of 21.1%. This incidence was slightly higher than 12.3% of pathogenic *E. coli* found in Kenya [20]. Conversely, the prevalence in our study was much higher than among 140 fecal samples collected from camels in the United Arab Emirates (4.3%) [21] and 3.8% among 600 fecal samples collected from camels in Nigeria [22].

Twelve *E. coli* isolates displayed four; four from serogroup O26 (33.3%), three each from O103 and O111 (25%), and two from O45 (16.7%). These results are somewhat consistent with Adamu *et al*. [22], who found that O26 was the main serotype in camel fecal samples (43.5%) with lesser incidence of O103 and O111 serotypes (17.4% and 13.0%, respectively); the O45 serogroup was not detected. Conversely, the O45 serotype was frequently encountered in camel feces by Hussni *et al*. [18]. The serotype O103 was detected along with other serotypes O2, O8, O83, and O120 in diarrheic camel calves [23].

*E. coli* isolates were more frequently resistant to vancomycin (75%) and ampicillin (66.6%). However, significant resistance was also observed to amoxicillin (58.3%) and streptomycin (50%). In contrast, isolates were found to be highly sensitive to ciprofloxacin, norfloxacin, and tetracycline (100%) and somewhat less sensitive to gentamycin and trimethoprim-sulfamethoxazole (83.3%). Previous evaluation of pathogenic *E. coli* isolates for antibiotic resistance varies. Bessalah *et al*. [24] indicated that *E. coli* isolates were sensitive to ciprofloxacin, gentamicin, amikacin, chloramphenicol, and ceftiofur and resistant to ampicillin and tetracycline. Further, *E. coli* isolates were 100% susceptible to ciprofloxacin, norfloxacin, cefotaxime, chloramphenicol, and polymyxin B [21].

**Figure-1:**
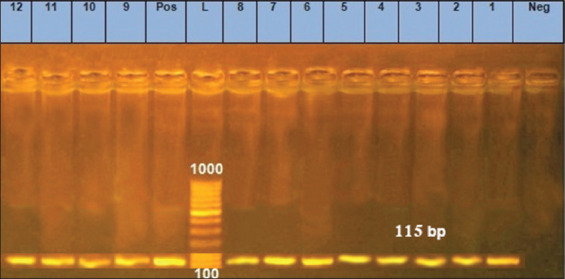
Uniplex polymerase chain reaction detection of structural gene in *Escherichia coli* isolates showing: L: 100 bp DNA ladder. Lanes 1-12: *E. coli* isolates. Lane pos.: Positive control; amplification of 115 bp represented *yai*O, lane neg.: Negative control.

*yai*O is a housekeeping gene of *E. coli*, and its detection confirms that isolates are *E. coli* strains. The intended 115 bp band was amplified in all 12 isolates to confirm *E. coli* (Figure-1). Molina *et al*. [9] reported that *yai*O amplification is highly specific for *E. coli*, with superior detection ability and non-redundancy with enzymatic methods.

**Figure-2:**
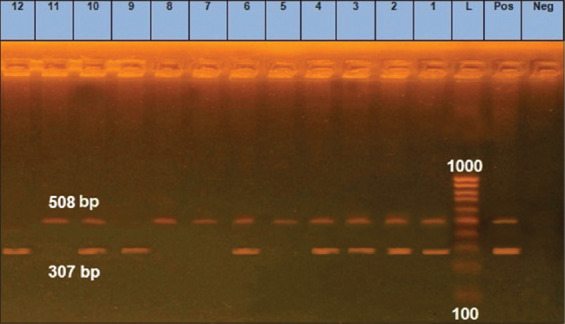
Diplex polymerase chain reaction detection of Shiga toxins genes in *Escherichia coli* isolates showing: L: 100 bp DNA ladder. Lane neg.: Negative control, lane pos.: Positive control; lanes 1-12 *E. coli* isolates (1-4; O26), (5-7; O103), (8-10; O111) and (11-12; O45), amplification of 307 bp represented *tra*T, 508 bp represented *fim*H.

The presence of the virulence genes is indicative of the pathogenicity of *E. coli* strains and is used to distinguish such strains from non-pathogenic bacteria. *tra*T is important for conjugal transfer in *E. coli*, encodes a complement resistance protein, and is responsible for preventing unproductive conjugation between bacteria carrying plasmids [10]. Moreover, adherence of *E. coli* to host receptors confers resistance to mechanical elimination and increases ­persistence. *fim*H is a major determinant encoding adhesins that target epithelial receptors; thus, the gene is crucial for *E. coli* colonization [11]. Results obtained from diplex PCR (Figure-2) recognized *tra*T and *fim*H genes in 8 and 10 isolates (75%, 83.3%), respectively. Moreover, all four isolates of serotype O26 harbored both virulence genes. The three isolates of serotype O103 carried *fim*H, but only one carried the *tra*T gene. Two of three isolates of serotype O111 carried *tra*T and *fim*H and the two O45 isolates harbored one gene each. Similar data were reported by Díaz-Jiménez *et al*. [25], who indicated that about 85% of *E. coli* isolates implicated diarrheic conditions carried *tra*T and *fim*H genes. Staji *et al*. [26] showed that the *tra*T gene is common enteropathogenic (EPEC) and enterohemorrhagic *E. coli* (EHEC) ink serotypes O26 and O111. Further, the *fim*H gene was expressed in enteropathogenic *E. coli* strains of zoonotic importance [27] and in multidrug-resistant diarrheagenic strains [28].

**Figure-3:**
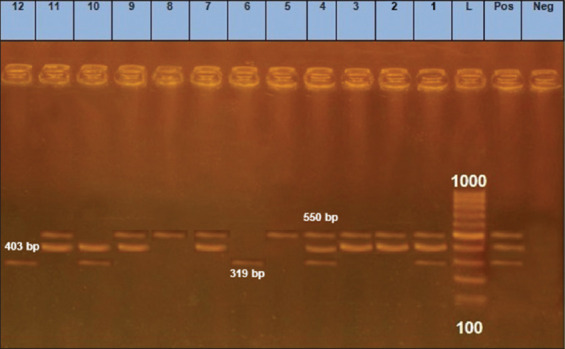
Multiplex polymerase chain reaction detection of antibiotic-resistant genes in *Escherichia coli* isolates showing: L: 100 bp DNA ladder. Lane neg.: Negative control, lane pos.: Positive control; lanes 1-12 *E. coli* isolates (1-4; O26), (5-7; O103), (8-10; O111), and (11-12; O45); amplification of 319 bp represented *aad*B, 403 bp represented *mph*A, and 550 bp represented *amp*C.

The emergence of multidrug-resistant strains is important for economic concerns as well as a public health [29]. The 12 *E. coli* isolates were checked for the presence of three prevalent antibiotic resistance genes (Figure-3). All 12 isolates harbored at least one gene. Nine carried *amp*C (75%), eight *mph*A (66.6%), and five *aad*B (41.7%). Two isolates harbored all three genes (25%), showed the O26 serotype. Five isolates carried two genes (41.7%). These data indicate that 7 isolates (58.3%) could be considered MDR. Interestingly, genotypic results are harmonized with the phenotype of one isolate; high and intermediate resistance to vancomycin, ampicillin, amoxicillin, and streptomycin. Multidrug resistance for this combination among DEC strains is frequently reported (80%, 94%, and 78.1%) [30-32], respectively.

Pathogenic and toxigenic *E. coli* are implicated in many disorders in animals, such as diarrhea and HC. The O26 serotype is the second most significant pathogenic serotype worldwide after O157:H7, since it produces toxins and causes zoonotic disease. The four O26 isolates were given more attention in the present study. These isolates were positive for *vt*1 and *vt*2, genes responsible for verocytotoxin expression. One isolate was positive for targeting *vt*1 at cycle 32, and three isolates were positive for targeting *vt*2. Previous reports also show an association between enterotoxigenic *E. coli* O26 strains and the presence of verotoxigenic genes [18,33].

DNA sequence was initially performed to establish identity to sequences in GenBank. *E. coli* O26 GH1, 2, 3, and 4 were selected for similarity to virulence *tra*T gene with genes in GenBank. Sequence alignments using NCBI BLASTP indicated close identity of *E. coli* O26 GH1 virulence *tra*T with *E. coli*_O157:H7_EC4115 (98.4%) and *E. coli* O26 GH2 with *E. coli* with *E. coli*_K_12_C3026 (97.5%). Both *E. coli* O26 GH3 and *E. coli* O26 GH4 showed identity with *E. coli*_O157:H7_TW14359 (97.7%). These results are supported by other investigations that indicate that *E. coli* O26 is an enterotoxigenic *E. coli* group related to *E*. coli O157 [34-36].

## Conclusion

In spite of camel has unique circumstances, but as other livestock suffer from different diseases, including diarrhea, caused by microbial agents. Enteropathogenic *E*. *coli* serotypes isolated from diarrheic neonatal camel calves displayed virulence and multidrug resistance, the ability to produce toxins, and possible importance for zoonotic disease transmission.

## Authors’ Contributions

MAS and AND designed the study. HAA and EAK collected samples and then performed the bacterial isolation and biochemical typing. ASH conducted serological typing. HAA and EAK performed DNA extraction and PCR. SFA and AND fulfilled the qRT-PCR, while EK and SFA implemented sequencing. MAS and AND analyzed the data, ASH and AND drafted the manuscript, revised, and finalized the manuscript for submission. All authors read and approved the final manuscript.
